# Genetic Abnormalities in Pancreatitis: An Update on Diagnosis, Clinical Features, and Treatment

**DOI:** 10.3390/diagnostics11010031

**Published:** 2020-12-26

**Authors:** Mitsuyoshi Suzuki, Kei Minowa, Satoshi Nakano, Hiroyuki Isayama, Toshiaki Shimizu

**Affiliations:** 1 Department of Pediatrics, Juntendo University Faculty of Medicine, Tokyo 113-8421, Japan; kminowa@juntendo.ac.jp (K.M.); snakano@juntendo.ac.jp (S.N.); tshimizu@juntedndo.ac.jp (T.S.); 2 Department of Gastroenterology, Juntendo University Graduate School of Medicine, Tokyo 113-8421, Japan; h-isayama@juntendo.ac.jp

**Keywords:** genetic mutations, chronic pancreatitis, pancreatic exocrine insufficiency, diabetes mellitus, pancreatic cancer

## Abstract

Several pancreatitis susceptibility genes have been identified to date. A relationship between a mutation in the cationic trypsinogen (protease serine 1, *PRSS1*) gene and hereditary pancreatitis (HP) was first identified in 1996. Currently, HP has been defined as either two or more individuals within a family exhibiting pancreatitis for two or more generations, or pancreatitis linked to mutation of the *PRSS1* gene. In 2000, a mutation in the serine protease inhibitor gene (*Kazal* type 1: *SPINK1*) was reported to be related to sporadic pancreatitis of unknown etiology. This paper reviews and summarizes the current published data on the pancreatitis susceptibility genes, mainly *PRSS1* and *SPINK1* genes, and introduces a diagnostic and therapeutic approach for dealing with patients with these gene mutations. Patients with these genetic predispositions, both children and adults, have often been initially diagnosed with idiopathic acute pancreatitis, in approximately 20–50% of pediatric cases and 28–80% of adult cases. In such patients, where the etiology is unknown, genetic testing, which requires pre-test and post-test genetic counselling, may prove helpful. Patients with chronic pancreatitis (CP) due to *SPINK1* gene mutation and HP patients have a potentially high risk of pancreatic exocrine insufficiency, diabetes mellitus, and, of particular importance, pancreatic cancer. Thus, these patients require careful long-term follow-up and management. Specifically, symptomatic CP patients often need endoscopic therapy or surgery, often following a step-up approach beginning with endoscopic therapy and progressing to surgery if necessary, which is similar to the therapeutic approach for patients with CP due to other etiologies. It is important that clinicians are aware of the characteristics of patients with pancreatitis susceptibility genetic abnormalities.

## 1. Introduction

When the patients with acute recurrent pancreatitis (ARP) or chronic pancreatitis (CP) showing autosomal dominant pattern of inheritance, these demographics have been characterized hereditary pancreatitis (HP). HP caused by cationic trypsinogen (serine protease 1; *PRSS1*) gene mutation results in ARP and CP in both children and adults with high penetrance [1,2]. Currently, HP has been defined as either, two or more individuals within a family exhibiting pancreatitis for two or more generations, or pancreatitis linked to mutation of the *PRSS1* gene. On the other hand, familial pancreatitis is a border term used to describe families in which pancreatitis occurs with a greater incidence than expected by chance alone in the general population. Patients with HP usually present clinically with recurrent bouts of acute pancreatitis (AP) in the first two decades of life. HP prevalence ranges depending on the region, from 0.3 to 0.57 per 100,000 people, according to national cohort data [3,4,5]. Progression to CP occurs in the late teenage years and early adult life. As damage to the pancreas progresses, malabsorption occurs due to pancreatic exocrine insufficiency, and diabetes mellitus develops due to pancreatic islet cell damage [3]. Several pancreatitis susceptibility genes have been identified so far (See “History” below). The mechanisms of developing pancreatitis due to genetic abnormalities are mainly classified into three genetic pathways, classified as: the trypsin-dependent pathway, misfolding and consequent endoplasmic reticulum stress, and related to the ductal pathway [6]. Compared to the other causes of ARP and CP, genetic pancreatitis has some unique clinical characteristics. Recently, several cohorts showed the natural history of patients with serine protease inhibitor gene (*Kazal* type 1: *SPINK1*) germline-related pancreatitis and HP caused by *PRSS1* gene mutation, indicating a high progression rate of pancreatic exocrine insufficiency and diabetes mellitus, as well as a significantly increased risk of pancreatic cancer [3,7]. This paper reviews and summarizes the currently published data on the genetic abnormalities of pancreatitis susceptibility genes, and introduces a diagnostic and therapeutic approach for patients with these gene mutations.

## 2. History

In 1952, Comfort and Steinberg [8] reported CP in six members of one family over three generations. This CP was assumed to be due to an autosomal dominant inheritance, and was called hereditary chronic relapsing pancreatitis. Later in 1962, Gross et al. [9] evaluated CP in 38 patients from five families. They proposed that HP be defined by the following characteristics: (1) three or more patients with pancreatitis within the same family; (2) onset at a young age; and (3) pancreatitis unrelated to excessive alcohol ingestion, gallstones, or trauma. HP is due to autosomal dominant inheritance with about 80% penetrance. A relationship between a mutation in the *PRSS1* gene and HP was identified in 1996 [1].

A few years before the *PRSS1* gene mutation was identified, the cystic fibrosis transmembrane conductance regulator (*CFTR*) gene, a causative gene of cystic fibrosis [10], was also reported to be a gene associated with ARP and CP [11,12]. In 2000, a mutation in the serine protease inhibitor gene (*Kazal* type 1: *SPINK1*) was reported to be related to chronic idiopathic pancreatitis of unknown cause [13]. Since then, genetic causes of pancreatitis, calcium-sensing receptor (*CASR*) in 2003 [14,15,16,17], chymotrypsin C in 2008 (*CTRC*) [18], claudin-2 (*CLDN2*) in 2012 [19], carboxypeptidase A1 (*CPA1*) in 2013 [20], carboxyl ester lipase (*CEL*) in 2015 [21], chymotrypsin B1 and B2 (*CTRB1*/*CTRB2*) in 2017 [22], pancreatic lipase (*PNLIP*) in 2019 [23], and transient receptor cation channel subfamily V member 6 gene (*TRPV6*) in 2020 [24] have also been reported to play key roles in the etiology of APR and CP. With respect to these genes, p.C563fsX673 and p.C596fsX695 in *CEL* gene [25], and p.S282P in *CAP1* gene [26] have also been determined as causative factors of HP, together with *PRSS1* gene. Conversely, *PRSS2* p.G191R variants, reported in 2006, mitigate intra-pancreatic trypsin activity and thereby protect against CP [27].

## 3. Evaluation of the Causes of ARP and CP

Alcohol abuse is a well-known risk factor for CP in adults, and thus this etiology accounts for more than 60% of cases of CP in adults [28]. However, only 2–5% of heavy drinkers develop CP, and it is assumed that not only alcohol, but also other factors such as genetic background are involved in a complex manner. A positive association between smoking tobacco and developing pancreatitis has also been recognized, independently of alcohol [29]. In children, the etiology is often drugs, infections, trauma, and anatomic anomalies, such as choledochal cysts and pancreaticobiliary maljunction [30,31,32].

**Figure 1:**
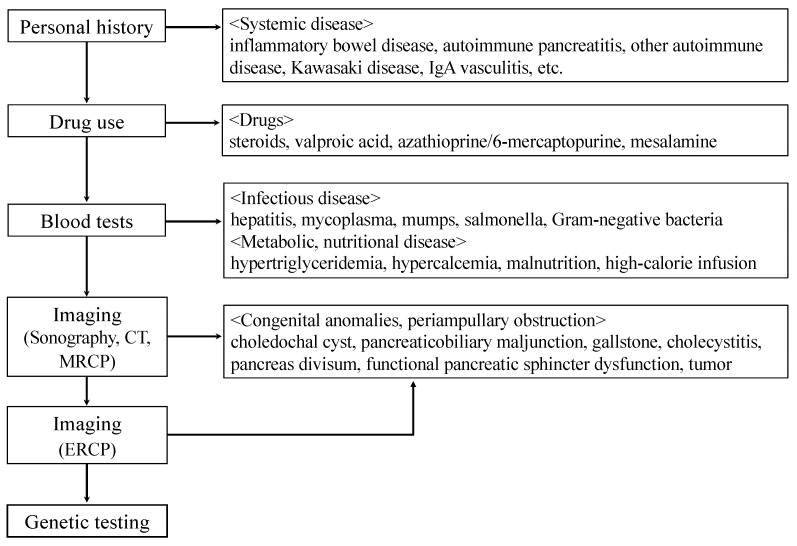
Identifying the cause of childhood acute pancreatitis (AP), acute recurrent pancreatitis (ARP), and chronic pancreatitis (CP).

Patients with these genetic predispositions, both children and adults, have often been initially diagnosed with idiopathic AP; in approximately 20–50% of pediatric cases [2,33] and 28–80% of adult cases [34]. In these ARP and CP patients, for whom the etiology is unknown, genetic testing may prove helpful (Figure 1). Genetic testing should be considered in patients who fulfill one or more of the criteria listed in Table 1 [35,36]. According to the European Registry of Hereditary Pancreatitis and Familial Pancreatic Cancer (EUROPAC), a diagnosis of HP can be made without genetic testing if the patient has both a personal history of pancreatitis and APR, or has CP been diagnosed in two first-degree relatives or in three or more second-degree relatives, spanning at least two generations [37]. Patients who undergo genetic testing should have genetic counseling prior to, and after, testing [36]. Unfortunately, even if pancreatitis-related genetic abnormalities are discovered at this time, there is no radical cure, such as gene therapy. However, proper diagnosis would also motivate patients and their families to continue treatment. Germline testing, currently for recommended etiologic mutations associated with pancreatitis, includes evaluation for pathogenic variants in the *CEL*, *CFTR*, *CPA1*, *CTRC*, *PRSS1*, and *SPINK1* genes in symptomatic individuals [29]. Asymptomatic testing can be considered in patients with first-degree relatives with known causative gene mutations, but it should be performed in the context of genetic counseling [36].

Clarifying the medical and personal history allows identification of the cause of pancreatitis, such as systemic diseases, drugs, and infectious diseases. Subsequent imaging studies, such as sonography, computed tomography (CT), and/or magnetic resonance cholangiopancreatography (MRCP), can detect anatomic anomalies and obstruction in the pancreaticobiliary system. Since MRCP has a low sensitivity for identifying pancreaticobiliary maljunction in young patients below about 2 years of age [38], MRCP should be repeated after a while or endoscopic retrograde cholangiopancreatography (ERCP) should be performed as necessary. 

## 4. Genetic Abnormalities

### 4.1. CFTR Gene

The *CFTR* gene has been identified as a causative gene of cystic fibrosis [10], and it is also reported to be a gene associated with pancreatitis [11,12]. About 1–4% of the overall cystic fibrosis population will have an episode of pancreatitis. Mutation causes a defect in the CFTR protein that causes abnormal HCO_3_^-^ transport, leading to defective pancreatic secretion [39]. As a result of impaired pancreatic juice alkalinization and water secretion, protein plugs form in the pancreas and/or pancreatic duct [40]. Regarding the relationship with CP, it has been reported that splicing efficiency and channel function decrease due to poly T polymorphism, TG repeat polymorphism, and p.Q1352H polymorphism [11,12,41]. Some *CFTR* mutations can be inherited in a complex-type pattern. The *CFTR* gene is considered to be a high-risk for developing CP when it is associated with other multiple mutations (complex heterozygotes mutation), especially *SPINK1* gene mutations [42,43,44].

### 4.2. PRSS1 Gene

Mutations in *PRSS,* which encodes cationic trypsinogen, the most abundant isoform of trypsinogen in human pancreatic juice, can occur. In 1996, the p.R122H in the *PRSS1* gene was first identified as a cause of HP [1]. In the following years, the p.N29I mutation was detected as a new mutation in HP patients [45]. The p.R122H mutation is the most common (~65%), followed by p.N29I mutation (~25%), and p.A16V. Increased trypsin levels are generated at the onset of pancreatitis, but not through the same biological mechanism. The p.R122H mutation (autolysis site) inhibits trypsin self-destruction, following increased trypsin stability and high level of trypsin in the pancreas, leading to pancreatic autodigestion and pancreatitis [46]. The p.A16V mutation (activation site) increases N-terminal processing of the trypsinogen activation peptide by CTRC, which in turn enhances autoactivation [46,47]. The p.N29I mutation affects both degradation and autoactivation in trypsinogen biochemistry [46].

Alternatively, a subset of *PRSS1* mutations can cause misfolding and endoplasmic reticulum stress [48]. In 2009, the p.R116C mutation found in HP families with incomplete penetrance was first reported as a cause of CP by a mis-folding-dependent pathway. Since then, several variants such as p.D100H, pC139F, p.K29N, p.S124F, and p.G208A have been reported, likely involving this pathway, but the detailed pathogenic mechanism is still unclear [49].

### 4.3. SPINK1 Gene

*SPINK1* encodes a pancreatic secretory trypsin inhibitor, and mutations interfere with the protective function, and predispose a person to pancreatitis, possibly via increased intrapancreatic trypsin activity. SPINK1, together with the protease inhibitors α_1_-antitrypsin and α_2_-macroglobulin, binds to activated trypsin and inhibits its activity. SPINK1 inhibits about 20% of total trypsin activity, and acts as a primary defense mechanism [50,51]. The trypsin binding site is encoded on exon 3. The most common mutation is the p.N34S mutation. According to the first repot by Witt et al., the p.N34S variant was found in 18/85 (21%) of children with idiopathic pancreatitis [13]. However, the p.N34S mutation is even present in 0–2% of otherwise healthy persons, suggesting that this mutation is thought to be a disease-modifying factor rather than causative factor, when additional risk factors for pancreatic inflammation such as alcohol, tobacco consumption, or genetic are present [51,52]. Actually, the p.N34S variation had no effect on the secretion of SPINK1 protein from transfected cells and trypsin inhibitory activity of the mutant protein was also unchanged [53,54]. Recently, the p.N34S mutation was found in 20% of patients carrying the functionally defective TRPV6 variants [24], suggesting that the combination of mutated TRPV6 and *SPINK1* p.N34S results in predisposition to pancreatitis, as well as the *CFTR* gene. The next most frequent mutation is the c.194 + 2T > C mutation, which has often been reported in Asian persons in Japan, China, and South Korea [55,56,57]. In the c.194 + 2T > C mutation, because exon 3 is skipped, due to a slicing aberration, trypsin activation cannot be inhibited, and pancreatitis occurs [58].

### 4.4. CTRC Gene

Following the finding that CTRC specifically degrades trypsin, the association between *CTRC* mutations and pancreatitis was investigated [18]. *CFTR* mutations have been shown to occur in 0.7% of healthy controls and 2.9% of adults with CP [18]. In a recent cohort from the International Study Group for Pediatric Pancreatitis: In Search of a Cure (INSPPIRE), it was reported that early-onset pancreatitis below 6 years of age was likely associated with genetic abnormalities, particularly *PRSS1* (43%) or *CTRC* (14%) mutations [2]. CTRC serves as a second lone defense against premature activation of trypsinogen isoforms [18]. Mutations of *CTRC* cause loss of function by several mechanisms, which include severe reduction of CTRC secretion (p.A73T), inactive CTRC (p.K247_R254del), promotion of degradation by trypsin (p.R254W), decreased CTRC activity (p.V235I), or decreased CTRC mRNA (p.G60 =) [18,59,60]. Of note, only *CTRC* pathogenic variants do not seem to cause CP, but rather they are seen in combination with other variants, such as *CTRC* or *SPINK1* mutations [18,43,60], or with anatomic anomalies of the pancreaticobiliary system [33,61].

### 4.5. CPA1 Gene

The mechanism by which a *CPA1* mutation confers an increased risk of pancreatitis involves misfolding-induced endoplasmic reticulum stress, rather than increased trypsin activity [20]. *CPA1* mutations with less than 20% apparent activity of the CPA1 protein have been observed to be significantly overrepresented in patients with CP. The functionally impaired *CPA1* variants with less than 20% functionality were found in 3.1% of non-alcoholic CP patients (29/944) and 0.1% of controls (5/3938) (*p < 0.01*). The most frequent functionally impaired variant was p.N256K, and it was observed in 0.7% (7/944) of patients and 0% (0/3938) of controls. The risk for pancreatitis was 38-fold greater in patients younger than 20 years old and 84-fold greater in patients younger than 10 years old. The associations between *CPA1* mutations and non-alcoholic CP patients have also been reported in additional cohorts from Europe (1.3% (8/600) and 0.4% (9/2432) (*p < 0.01*)), India (2.5% (6/239) and 0.3% (1/340) (*p < 0.05*)), and Japan (2.0% (5/247) and 0% (0/341) (*p < 0.05*)) [20].

### 4.6. TRPV6 Gene

TRPV6 is a member of the transient receptor potential vanilloid ion channel superfamily [62]. TRPV6 promotes high Ca^2+^ entry in absorptive and secretory tissues. It is mainly expressed in Ca^2 +^ -transporting epithelia. In the pancreas, TRPV6 expression is nearly 6-times higher in ductal cells than in acinar cells [63]. More recently, Masamune et al. reported that impaired Ca^2+^ uptake caused by *TRPV6* variants was associated with early-onset CP [24]. Interestingly, 6 out of 30 (20%) patients with functionally defective *TRPV6* variants were trans-heterozygous for *SPINK1* p.N34S [24], indicating that CP is a complex multigenic disease, and a cumulative genetic handicap seems to be crucial for the development of early-onset CP.

### 4.7. Others

CASR, first characterized in the bovine parathyroid [64], expressed in the pancreas can respond to high calcium concentrations in the pancreatic juice by increasing ductal fluid secretion, thereby preventing stone formation and pancreatitis [65]. An association between developing CP and variants in the *CASR* gene has been reported [14,15,16,17], but the evidence remains uncertain.

PRSS2 is another major trypsinogen isoform constituting the bulk of secreted trypsinogen in humans [66]. No pathogenic *PRSS2* variants have been identified in HP and sporadic pancreatitis [27]. The variant p.G191R introduces a trypsin cut site anionic trypsin, which reduces the overall activity of *PRSS2*, indicating that this variant confers protection from CP [27].

CLDN2 is expressed in the proximal pancreatic duct and promotes H_2_O and Na^+^ transport to counter Cl^-^ and HCO_3_^-^ secretion through CFTR [19]. *CLDN2* mutations, an X-chromosome locus gene, were found to be associated with alcohol-related and sporadic pancreatitis [19]. Since men are hemizygous for the X chromosome, the risk appears dominant, whereas it is inherited as a recessive pattern in women.

Mutations in CEL cause maturity-onset diabetes of the young type 8 (MODY8), as well as pancreatic exocrine dysfunction [25]. A hybrid *CEL* allele (*CEL-HYB1*), formed by nonallelic homologous recombination between *CEL* and its adjacent pseudo-gene *CELP*, was enriched approximately 5-fold in patients with idiopathic CP [21]. This hybrid protein was poorly secreted due to intracellular retention, leading to endoplasmic reticulum stress and apoptosis in an in vitro experiment [21].

The changes in the balance of chymotrypsin isoforms, namely inversion at the CTRB1 and CTRB2 locus, that affect trypsin degradation slightly increased the risk of alcoholic and non-alcoholic CP [22,67]. In addition, several variants of *PNLIP* gene, particularly p.F300L, were associated with early onset and non-alcoholic CP in the European population, but the mechanism remains unclear [23].

## 5. Clinical Features of *PRSS1* and *SPINK1* Gene Mutation-Related Pancreatitis

### 5.1. Age at Symptom Onset, Pancreatic Exocrine Insufficiency, and Diabetes Mellitus

ARP is frequently a precursor to CP, and both are thought to be on the same disease continuum. In particular, genetic factors are associated with early progression of ARP to CP [5,24,37,68]. Most patients developed their first symptoms before they were 30 years old. HP patients are known to have a higher future risk of developing pancreatic exocrine insufficiency and diabetes mellitus [2]. In the EUROPAC study that enrolled 418 HP patients in 112 families (52% families had the *PRSS1* p.R122H, 21% had the *PRSS1* p.N29I, and 19% had no *PRSS1* mutations) [37], the median age at symptom onset was 10 years in p.R122H, and 14 years in p.N29I. Pancreatic exocrine insufficiency and diabetes mellitus developed in 37% and 48% of patients at 50 years of age, and 60% and 70% at 70 years of age, respectively. The average age from onset to pancreatic exocrine insufficiency and diabetes mellitus was as long as 53 years. In a French study that enrolled 200 HP patients in 78 families, *PRSS1* mutations were found in 68% (p.R122H 78%, p.N29I 12%, others 10%), and the median age at first symptom onset was 10 years [5]. Pancreatic exocrine insufficiency developed in 34% of patients (median age of occurrence, 29 years), and diabetes mellitus in 26% of patients (median age of occurrence, 38 years) [5].

Previous studies have assessed the association between mutation type and disease phenotype [37], but the results were inconsistent. A recent Japanese nationwide survey enrolled 271 HP patients from 100 families (*PRSS1* p.R122H 33%, *PRSS1* p.N29I 8%, *SPINK1* p.N34S 22%, *SPINK1* c.194 + 2T > C 14%, *SPINK1* p.P45S 1%) and showed that the mean age at symptom onset was 12.3 years in patients with *PRSS1* mutations, and 20.0 years with *SPINK1* mutations [3]. The cumulative rates of pancreatic exocrine insufficiency and diabetes mellitus in both patients with *PRSS1* and *SPINK1* mutations were 16.1% and 5.5% at 20 years of age, and 45.3% and 28.2% at 40 years of age, respectively [3]. In this survey, HP was defined in ARP and/or CP patients with a family history of two or more affected patients, irrespective of generation, with at least one of the patients having an unknown etiology, and in the case of siblings only, age at onset before 40 years was needed to define HP [3]; thus, patients with *SPINK1* gene mutations could be included in the category of HP.

More recently, Muller et al. [7] focused only on 209 patients with *SPINK1* mutations, and reviewed their prognosis. The median onset of symptoms was 20.1 years. The cumulative rates of pancreatic exocrine insufficiency and diabetes mellitus were 5.3% and 7.8%, 14.7% and 13.4%, 28.3% and 26.3%, 52.4%, and 43.4% at 30, 40, 50, and 60 years of age, respectively [7]. These results supported the concept that patients with *PRSS1* mutations developed pancreatic exocrine insufficiency and diabetes mellitus earlier than those without *PRSS1* or *SPINK1* mutations; thus, *PRSS1*-associated HP patients show severe phenotypes.

### 5.2. Pancreatic Cancer

It is well known that CP patients with *PRSS1* or *SPINK1* gene mutations belong to a high-risk group for pancreatic cancer, with an estimated risk 53–87 times higher than the normal population [37,69,70]. In the EUROPAC study, the overall cumulative risk of pancreatic cancer was 0% to 30 years, 0.5% at 40 years of age, 3.4% at 50 years, 9.8% at 60 years, 18.8% at 70 years, and 33.3% at 80 years [37]. In a French cohort of HP patients, the cumulative rates of pancreatic cancer diagnosis at 50, 60, and 75 years of age were 10.0%, 18.7%, and 53.5%, respectively [69]. In a recent Japanese HP cohort, the cumulative rates at 40, 60, and 70 years of age were 2.8%, 10.8%, and 22.8%, respectively [3]. From the data of a cohort focused on patients with *SPINK1* mutations, the cancer risk was 12-times higher in patients than in controls, and the cumulative rates of pancreatic cancer before 50, 60, 70, and 80 years of age were 0.8%, 11.9%, 27.7%, and 51.8%, respectively [7]. Overall, the cumulative rates of pancreatic cancer at 40, 50, 60, 70, and 80 years of age were 0.5–2.8%, 0.8–10.0%, 9.8–18.7%, 7.2–40.0%, and 33.3–51.8%, respectively. Thus, development of pancreatic cancer was not restricted to *PRSS1*-asociated HP, but was also found in patients with *SPINK1* germline mutations, suggesting that long-term inflammation and existing CP involving hyperplasia and metaplasia of the pancreatic duct epithelium, not the mutations alone, increase the risk of pancreatic cancer.

## 6. Treatment

Patients with genetic abnormalities are usually managed in the same way as patients who present with AP, ARP, and CP of other etiologies. The initial treatment for AP is to withhold oral intake of food or fluid to prevent stimulation of pancreatic exocrine secretions, allowing the pancreas to rest. Fluid and electrolyte supplementation and treatment to relieve pain and prevent infection are provided. AP is known to be associated with the activation of clotting and the degree of this activation is closely related to the severity of AP [71,72]. Heparin in complex with antithrombin prevents coagulation by inhibiting proteases involved in the coagulation cascade, such as activated forms of factor XII, XI, IX, X, VII, and thrombin [73]. Furthermore, heparin directly and indirectly inhibits the activity of pancreatic digestive enzymes, such as trypsin and chymotrypsin [74,75], as well as inhibiting the conversion of trypsinogen to active trypsin [76]. Considering the mechanism of developing pancreatitis due to *PRSS1* mutations, these data suggest that anticoagulants, especially heparin, may be useful in the prevention and/or treatment of HP.

Symptomatic CP patients often require endoscopic treatment or surgery. Endoscopic treatments were performed in 16.0% (32/200) [5] and 22.6% (35/155) [3] of HP patients. Surgical procedures were required in 19.4% (81/417) [37], 16.0% (32/200) [5], and 17.4% (27/155) [3] of HP patients. In the EUROPAC study, the cumulative risk of pancreatic resection was 0.6% to 10 years of age, 2.5% at 20 years, 8.3% at 30 years, 11.4% at 40 years, 17.5% at 50 years, and 21.5% at 70 years [37]. Only a cohort from Japan showed the cumulative risks of both endoscopic treatment and surgical procedures, which were 2.0% and 2.8% at 10 years of age, 12.3% and 10.1% at 20 years, 29.0% and 25.2% at 40 years, and 43.0% and 28.1% at 60 years, respectively [3]. Recently, the effectiveness of total pancreatectomy with autotransplantation (TPIAT) has been well described in both adults and children with CP unresponsive to medical and endoscopic treatments [77,78,79]; which is also more likely to be performed if underlying genetic risk has been demonstrated [77,78].

Currently, since endoscopic therapy of CP is an effective treatment in adults, and the evidence of its efficacy for pediatric populations has been growing [80,81], a step-up strategy has become increasingly standard and more commonly applied for the treatment of both adult and pediatric CP patients [82,83,84]. This strategy starts with endoscopic treatment and progresses to surgery if endoscopic therapy fails or proves technically impossible [84]. Treatment involves endoscopic retrograde cholangiopancreatography with pancreatic sphincterotomy, pancreatic stent placement, and stone removal. Various ingenious techniques and devices have been developed for endoscopic treatment, such as multiple plastic stents placement or fully-covered self-expandable metallic stent placement [85,86,87].

**Figure 2:**
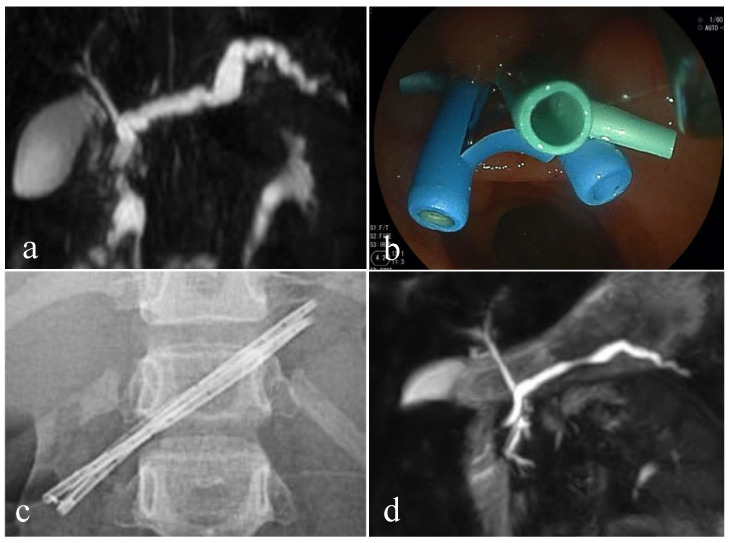
Multiple plastic stent placement therapy for children with hereditary pancreatitis. Magnetic resonance cholangiopancreatography (MRCP) before endoscopic treatment. The main pancreatic duct is meandering and markedly dilated (**a**). Endoscopic view of placement of 3 plastic stents from the minor papilla (**b**). Abdominal X-ray shows 3 plastic stents placed in the main pancreatic duct (**c**). MRCP 16 months after plastic stent removal. Dilation of the main pancreatic duct is improved (**d**).

Pediatric cases of *SPINK1* germline mutation-related CP who underwent endoscopic treatment are shown in Figure 2. An 8-year-old girl, with compound heterozygous for *SPINK1* p.N34N/S and c.194 + 2T > C mutations, suffered from recurrent pancreatitis with main pancreatic duct dilation (Figure 2a). Endoscopic pancreatic stenting with a 7 Fr plastic stent was performed across the minor papilla after sphincterotomy to prevent recurrent pancreatitis. Major papilla cannulation failed because of pancreas divisum, or obstruction of Wirsung’s duct due to chronic inflammation. To resolve the pancreatic ductal stricture, multiple plastic stents placement was planned, by exchanging the plastic stent about every 3 months, gradually increasing the size and number of plastic stents, two times (7 Fr + 5 Fr) and two times (7 Fr + 7 Fr + 5 Fr) (Figure 2b,c). Multiple plastic stents were successfully removed with improvement of the main pancreatic duct stricture 18 months after the initial stenting. The main pancreatic duct dilation was improved, confirmed by magnetic resonance cholangiopancreatography, without episodes of recurrent pancreatitis 16 months after the stent removal (Figure 2d).

## 7. Conclusions

Clinicians need to know the characteristics of ARP and CP with genetic abnormalities. Genetic testing for patients with unknown etiology is useful by analyzing *CEL*, *CFTR*, *CPA1*, CTRC, *PRSS1*, and *SPINK1* genes, after the major causes of AR and CP have been excluded. In addition, testing for protective variants of *PRSS2* gene is not useful clinically. The clinical usefulness of testing mutations of the genes encoding *CLNN2*, *CTRB1*, *CTRB2,* and *PNLIP* is limited due to their high frequency and narrow range of clinical symptoms. Genetic counseling prior to and after testing is required in all patients. Clinicians should carefully follow ARP and CP patients with genetic mutations, since they have a potentially high risk of developing pancreatic exocrine insufficiency, diabetes mellitus, and pancreatic cancer.

## Figures and Tables

**Table 1:** Criteria for genetic testing for hereditary pancreatitis.

Consider When Patients Meet One or More of the Following Criteria:	
1	A family history of idiopathic CP, ARP, or childhood pancreatitis
2	Relatives with known mutations associated with HP
3	Unexpected pancreatitis in a child
4	Idiopathic CP in patients < 25 years old
5	ARP of uncertain etiology
6	Patients who meet criteria for participation in approved research projects

HP: hereditary pancreatitis, CP: chronic pancreatitis, ARP: acute recurrent pancreatitis.

## Data Availability

The data that support the findings of this study are available from the corresponding author, M.S., upon reasonable request.
